# Overweight and abdominal obesity as determinants of undiagnosed diabetes and pre-diabetes in Bangladesh

**DOI:** 10.1186/s40608-016-0099-z

**Published:** 2016-03-18

**Authors:** Dewan S. Alam, Shamim H. Talukder, Muhammad Ashique Haider Chowdhury, Ali Tanweer Siddiquee, Shyfuddin Ahmed, Sonia Pervin, Sushmita Khan, Khaled Hasan, Tracey L. P. Koehlmoos, Louis W. Niessen

**Affiliations:** School of Kinesiology and Health Science, Faculty of Health York University, Room 362, Stong College, 4700 Keele St, Toronto, ON M3J 1P3 Canada; Eminence, Hena Nibash, 3/6, Asad Avenue, Mohammadpur, Dhaka 1207 Bangladesh; Centre for Control of Chronic Diseases, icddr,b, Mohakhali, Dhaka, Bangladesh; Department of Preventive Medicine and Biostatistics, Uniformed Services University of the Health Sciences, 4301 Jones Bridge Road, Bethesda, Maryland, 20814-4799 USA; Centre for Apllied Health Research and Delivery, Liverpool School of Tropical Medicine, Pembroke Place, L3 6PQ Liverpool, UK

**Keywords:** Diabetes, Pre-diabetes, Prevalance, Determinants, Screening, Urban, Rural, Bangladesh

## Abstract

**Background:**

Type 2 diabetes and pre-diabetes are an increasing pandemic globally and often remain undiagnosed long after onset in low-income settings. The objective of this study is to assess the determinants and prevalence of undiagnosed diabetes and pre-diabetes among adults in Bangladesh.

**Methods:**

In an exploratory study, we performed oral glucose tolerance test on 1243 adults ≥20 years of age from urban Mirpur, Dhaka (*n* = 518) and rural Matlab, Chandpur (*n* = 725) who had never been diagnosed with diabetes or pre-diabetes. We collected data on socioeconomic, demographic, past medical history, physical activity, and measured weight, height, waist and hip circumferences, and blood pressure. Risk factors associated with undiagnosed diabetes and pre-diabetes were examined using a multiple logistic regression model.

**Results:**

Overall prevalence of diabetes and pre-diabetes was 6.6 % (95 % CI 5.3, 8.1) and 16.6 % (14.5, 18.7) respectively, with both being significantly higher in urban than rural populations (diabetes 12.2 % vs 2.6 % respectively, *p* < 0.000; pre-diabetes 21.2 % vs 13.2 %, *p* < 0.001). After adjustment the variables, urban residence (OR 2.5 [95 % CI 1.02, 5.9]), age group 40–59 y (2.9 [1.7–5.2]), ≥60 y (8.1 [2.8–23.8]), overweight (2.2 [1.3–3.9]), abdominal obesity (3.3 [1.8–6.0]) and high WHR 5.6 (2.7–11.9) were all significant predictors of diabetes. Significant predictors of pre-diabetes included age group 40–59 (1.6 [1.1–2.2]), female sex (1.5 [1.0–2.2]), abdominal obesity (1.7 [1.2–2.4]) and high WHR (1.6 [1.2–2.3]).

**Conclusion:**

Both overweight and abdominal obesity contribute to the hidden public health threat of undiagnosed diabetes and pre-diabetes. Awareness raising and screening of high risk groups combined with a tailored approach are essential for halting the epidemic of diabetes and pre-diabetes in Bangladesh.

## Background

Type 2 diabetes prevalence is reaching epidemic proportions in many countries across the world [1]. Global rise in type 2 diabetes (T2D) is projected to be disproportionately higher in low-income countries and will especially affect adults in their working ages [2]. Bangladesh is one of the top 10 high-burden diabetes countries worldwide with an estimated 8.4 million people with diabetes and another 7.8 million with pre-diabetes, an interim hyperglycaemic condition above normal but below the cut-off for diabetes [1]. It is projected that Bangladesh will experience the highest growth in diabetes population and will rank 5th in the world with 16.8 million adults with diabetes by 2030 [3]. A recent metanalysis of Bangladeshi literature reported an increasing trend in diabetes prevalence from 3.8 % in late 1990s to 9 % in 2006–2010 [4]. This is likely related to an increasing and shifting towards overnutrition from double burden of under- and overnutrition in Bangladesh [5].

Both diabetes and pre-diabetes are established cardiovascular risk factors [6]. The diabetes-associated cardio-vascular disease burden has been reported to be high in Asian populations [7]. A large proportion of people with diabetes and pre-diabetes remain undiagnosed for a long time, and are often diagnosed only when complications develop or during opportunistic screening while visiting health care facilities for other medical conditions [8]. A recent study among people with newly diagnosed type 2 diabetes in Bangladesh reported 84 % of patients had poor to average basic knowledge about the disease [9]. Complications due to hyperglycaemia may also develop during the pre-diabetes period [10, 11]. Although about 5 % of individuals with pre-diabetes advance into diabetes annually [12], timely diagnosis of individuals with prediabetes followed by lifestyle intervention can potentially prevent this conversion up to −58 % [13]. Screening and treating pre-diabetes is shown to be cost effective, in particular when combined with multi-factorial approach including lifestyle interventions [14, 15]. However, the very low awareness of the rising epidemic of diabetes in Bangladesh remains a big challenge. A recent study in Bangladesh reported that only 41 % of diabetes patients were aware of their condition [16].

With accelerating epidemiologic and demographic transitions combined with increasing and rapid urbanization and along with changes in lifestyle, diet, and physical activity in Bangladesh [17], there is an urgent need for continuous monitoring of the diabetes burden using rigorous diagnostic methods and the study of risk factors to develop effective control strategies. This population-based exploratory study measures the determinants and prevalance of both undiagnosed diabtes and pre-diabetes in Bangladesh and identifies high-risk groups.

## Methods

**Fig. 1:**
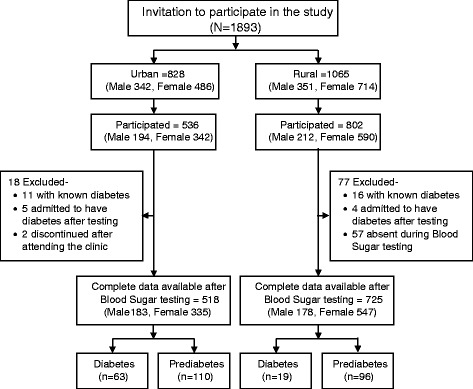
Study partcipation flow chart

Between March and October 2009, we conducted this population-based cross-sectional exploratory study in urban Mirpur, Dhaka District and in rural Matlab in Chandpur District, Bangladesh. The study population consisted of males and non-pregnant females ≥ 20 years of age who had never been diagnosed with diabetes or advised of having a blood glucose abnormality by a medical practitioner. The Matlab participants (*n* = 1065) included all available, eligible and consenting individuals from the population database of three villages selected from the Health Demographic Surveillance System (HDSS) of International Centre for Diarrhoeal Diseases Reseaerch, Bangladesh (icddr,b) at Matlab. The population database has been maintained by icddr,b since 1963 [18]. The urban Mirpur, Dhaka participants were selected from middle class settlement at Mirpur, Dhaka where another population database maintained by Eminence, a national Non-Government Organization (NGO).. All available, eligible and consenting individuals (*n* = 828) were invited to participate in the study. In both study areas, we conducted a door to door visit to confirm the availability of the selected participants and invited for a clinic visit for an interview, physical measurements and oral glucose tolerance test (OGTT). Individuals with known diabetes, or those unwilling or unable to participate, or unable to provide informed consent were excluded. An informed written consent was obtained from each participant before enrollment. The study was approved by the Ethical Review Committee (ERC) of International Centre for Diarrhoeal Diseases Research, Bangladesh (icddr,b). Fig. 1 presents a participation flow diagram.

We collected individual-level data on household socioeconomic status, demographics, family history, medical history, and lifestyle related variables using a pre-coded structured questionnaire administered by trained interviewers. We collected dietary data, with particular emphasis on fruit and vegetable consumption, using a food frequency questionnaire. All questionnaires were pretested before actual data collection and modified questions for clearity reasons based on the feedback from the field research staff. We also collected physical activity data through questionnaire and summarized average daily and weekly activity patterns with major categories whether the participant performed 150 min or longer moderate to heavy physical activity during the last 1 week. We measured weight, height, waist circumference (WC) and hip circumference (HC)s, and blood pressure (BP). Weight was measured to the nearest 100 g using Tanita (Model No. HD 318) digital weighing scale and height was measured to the nearest 0.1 cm using a locally constructed height stick. World Health Organization (WHO) definitions of threshold values were used for classifying BMI, waist circumference, and waist-hip ratio. BP was measured using Omron M10 automatic digital sphygmomenometer. We allowed 10 min rest before measuring BP and measured the BP three times at 5 minutes interval on the left arm in a sitting position with the arm supported at the level of the heart. The first BP measurement was discarded and mean value of the last two measurements was considered as the participant’s BP.

### Oral Glucose Tolerance Testing (OGTT)

We invited selected and consented participants to visit to the field clinic for interview, OGTT, and physical measurements. The participants were advised to adhere to their usual diet, avoid vigorous physical activity for at least 48 h prior to the scheduled clinic visit, and attend the clinic in the morning after an overnight fasting for 10–12 h. Using finger prick blood in a HemoCue™ 201 glucometer (HemoCue™ Sweden) we measured fasting capillary blood glucose concentration followed 2 hours later by drinking 75 g anhydrous glucose disolved in 200 ml water. HemoCue™ 201 glucometer (HemoCue™ Sweden) is a validated instrument against laboratory value of plasma glucose [19] which provides a digital display equivalent to plasma glucose concentration.

### Outcome definitions

Diabetes was defined as fasting blood glucose concentration ≥7.0 mmol/L, or ≥11.1 mmol/L at 2 hours after oral glucose challenge [20]. Individuals were considered having pre-diabetes if they had Impaired Fasting Glucose (IFG), indicated by a fasting blood glucose concentration between 5.6 mmol/L and 6.9 mmol/L, or Impaired Glucose Tolerance (IGT), indicated by a blood glucose concentration between 7.8 mmol/L and 11.0 mmol/L at 2 hours after oral glucose challenge [20].

### Data analysis

Senior research assistants scrutinized all completed questionnaires during the clinic visits for errors. Data were then entered in a computer using Microsoft Access with built in range and consistency checks. Distributions and type of distribution of all continuous variables including normality of distribution were examined to identify the outliers and extreme values. Data were summarized and presented as mean and standard deviation for the continuous variables and as frequency and percentages for the categorical variables. Linear relationship between independent vaiables was examined by correlation analysis. Student’s *t*-test was used to compare the means of continuous variables, z-test was used for comparing the proportions, and chi-square test was used for the discrete data. Initial association between risk factors and outcome (diabetes/pre-diabetes) was examined controling for age and sex only followed by a multiple logistic regression model to identify determinants of diabetes and pre-diabetes .. In the multivariate analysis, Body Mass Index (BMI), waist circumference, and waist hip ratio, were entered separately into the models to avoid possible multi-colinearity of these highly correlated variables. Associated risk for each of the determinants was expressed as an odds ratio (OR) with a 95 % confidence interval. A *p*-value <0.05 was considered statistically significant.

## Results

Table 1 presents the characteristics of study participants. In Mirpur Dhaka, out of available 828 adults invited, 536 participated. A total of 11 with known diabetes and five others who admitted their diabetes status after testing were excluded. Two additional participants discontinued after attending the clinic. Completed questionnaires were available for 518 participants with an overall response rate of 62 %. In Matlab, of 1065 individuals invited, 828 attended the clinic of whom 77 were excluded (16 known diabetes, four admitted their diabetes status after the blood test and 57 were not available during blood sugar testing). Eventually, complete data were available for 725 individuals constituting a response rate of 68 %.

**Table 1:** Characteristics of the study participants

Variable (Unit)		Total *n* = 1243	Urban			Rural		
			Total	Male	Female	Total	Male	Female
			*n* = 518	*n* = 183	*n* = 335	*n* = 725	*n* = 178	547
Age (Year)	Mean (SD) ^a^	41.5 (8.2)	41.2 (9.9)	44.4 (10.1)	39.5 (9.3)	41.6 (6.7)	42.5 (7.0)	41.3 (6.6)
	Age Group ^d,e^							
	20-39 Year (%)	42.9	47.1	36.6	52.8	39.9	36.0	41.1
	40-59 Year (%)	54.6	47.1	53.6	43.6	60.0	64.0	58.7
	60 Year or Above (%)	2.5	5.8	9.8	3.6	0.1	0	0.2
Male (%)		29.0	35.3			24.6		
Education	Median (25th–75th) ^c,a^	6 (0–10)	10 (8–14)	12 (10–15)	10 (8–12)	4 (0–6)	4 (0–7)	4 (0–5)
	Level of Education ^d,e^							
	Illiterate (%)	28.7	7.6	3.3	9.9	43.7	43.8	43.7
	Primary (%)	20.5	6.2	.5	9.3	30.6	27.0	31.8
	Secondary (%)	30.5	40.7	28.0	47.6	23.3	25.8	22.5
	Post secondary (%)	20.3	45.5	68.1	33.2	2.3	3.4	2.0
Household income (Taka^b^ ,000)	Median (25th-75th) ^c,a^	10 (5–15)	18 (10–25)	18 (10–28)	16 (10–25)	6 (4–10)	5 (4–8)	6 (4–10)
	Income Group ^d^							
	Low: ≤6,000 Taka/mo (%)	38.4	5.8	6.0	5.7	61.7	68.0	59.6
	Medium: 6,001–12,000/mo (%)	28.6	28.5	28.6	28.4	28.7	25.8	29.6
	High: >12,000 (%)	33.0	65.7	65.4	65.9	9.7	6.2	10.8
Boby Mass Index (BMI) (kg/m2)	Mean (SD) ^c,a^	23.0 (4.5)	25.9 (4.2)	24.7 (3.4)	26.6 (4.5)	21.0 (3.5)	20.3 (3.0)	21.2 (3.7)
	BMI Category ^d,e^							
	Underweight (≤18.49 kg/m^2^) (%)	50.9	38.2	50.8	31.3	60.0	60.7	59.8
	Normal (18.50–24.99 kg/m^2^) (%)	16.9	3.3	3.3	3.3	26.6	32.0	24.9
	Overwt/Obese (> = 25 kg/m^2^) (%)	32.2	58.5	45.9	65.4	13.4	7.3	15.4
Waist circumference (cm)	Mean (SD) ^c,a^	78.6 (11.3)	85.8 (10.1)	88.2 (7.9)	88.4 (10.9)	73.6 (9.1)	75.0 (9.3)	73.1 (9.0)
	Abdominal Obesity ^d,e^							
	Normal (<90 cm M,<80 cm F) (%)	62.8	37.8	54.1	29.0	80.6	92.7	76.6
	Anbdominally Obese (≥90 cm M,≥80 cm F) (%)	37.2	62.2	45.9	71.0	19.4	7.3	23.4
Waist-to-Hip Ratio ^d,e^	Normal (<0.90 M,<0.85 F) (%)	48.1	26.6	12.0	34.6	63.4	58.4	65.1
	High (≥0.90 M, ≥0.85 F) (%)	51.9	73.4	88.0	65.4	36.6	41.6	34.9
Vegetable/Fruit Consumption	2–3Serving/day (%)^d^	79.2	70.6	68.6	71.6	80.5	84.8	79.1
	4–5Serving/day (%)^d^	4.3	22.9	14.3	27.0	1.5	2.8	1.1
Level of Physical Activity^d^	Low (<=150 min/week) (%)	68.1	97.5	97.8	97.3	47.0	36.0	50.6
	High (>150 min/week) (%)	31.9	2.5	2.2	2.7	53.0	64.0	49.4
Glucose (mmol/L)	Fasting Mean (SD)^c^	5.1 (1.3)	5.4 (1.7)	5.3 (1.3)	5.6 (1.9)	4.8 (0.9)	4.7 (1.0)	4.9 (0.9)
	2 hr after glucose Mean (SD) ^c,a^	7.2 (2.7)	7.9 (3.4)	7.5 (3.2)	8.1 (3.5)	6.7 (2.0)	6.2 (2.0)	6.8 (2.0)
	Metabolic Status^c^							
	Diabetes (%)	6.6	12.2	13.1	11.6	2.6	2.2	2.7
	Prediabetes (%)	16.6	21.2	19.1	22.4	13.2	9.0	14.6
	Normal (%)	76.8	66.6	67.8	66.0	84.1	88.8	82.6

^a^Significant difference between male and female at <0.05 level

^b^Bangladesh Taka 76.00 = USD 1.00

^c^Significant difference between rural and urban residence at <0.05 level

^d^Significant association with area of residence (rural and urban) at <0.05 level

^e^Significant association with sex of the respondents at <0.05 level

The mean (SD) age of the participants was 41.5 (8.1) years with an average BMI of 23.0 (4.5) kg/m^2^. The mean BMI was significantly higher among urban than rural residents (26.0 kg/m^2^ vs 21.0 kg/m^2^; *p* < 0.01) as was the prevalence of overweight (58.5 % vs 13.4 %, *p* < 0.01). Overall, 6.6 % (95 % CI 5.3, 8.1) participants had diabetes, 16.6 % (95 % CI 14.5, 18.7) had pre-diabetes, and 1.3 % had isolated impaired fasting glucose (IFG). In the preliminary analysis (age and sex adjusted only), urban residence, higher education, higher household income, and overweight, abdominal obesity, high waist hip ratio,were associated with higher probability of both undiagnosed diabetes and pre-diabetes. Underweight and higher physical activity were associated with lower odds for diabetes and pre-diabetes. High vegetable consumption was significantly associated with lower odds for prediabetes (Table 2).

**Table 2:** Age and sex adjusted Odds Ratio (OR) for diabetes and prediabetes

Variable	Diabetes		Prediabetes	
	OR (95 % CI)	*P*-Value	OR (95 % CI)	*P*-Value
Area of Residence				
Rural	1.00		1.00	
Urban	5.6 (3.8–11.4)	0.000	2.2 (1.6–3.0)	0.000
Education				
Illiterate	1.00		1.00	
Primary	0.7 (0.3–1.9)	0.541	1.4 (0.8–2.2)	0.200
Secondary	4.5 (2.3–8.8)	0.000	2.3 (1.5–3.5)	0.000
Post secondary	3.8 (1.7–8.1)	0.001	2.8 (1.8–4.6)	0.000
Household Income (Taka)^a^				
Low (≤6,000 Taka/mo)	1.00		1.00	
Medium (6,000–12,000 Taka/mo)	3.2 (1.5–6.8)	0.002	1.8 (1.2–2.7)	0.003 ^a^
High (>12,000 Taka/mo)	6.3 (3.2–12.5)	0.000	2.2 (1.5–3.1)	0.000 ^a^
Body Mass Index (BMI)				
Normal (18.50–24.99 kg/m^2^)	1.00		1.00	
Underweight (≤18.49 kg/m^2^)	0.3 (0.1–0.9)	0.05	0.6 (0.4–1.02)	0.062
Overweight (≥25 kg/m^2^)	4 (2.4–6.6)	0.000	1.8 (1.3–2.5)	0.000
Waist to Hip Ratio (WHR)				
Normal (<0.90 M,<0.85 F)	1.00		1.00	
High (≥0.90 M, ≥0.85 F)	9.1 (4.4–18.5)	0.000	2.03 (1.5–2.8)	0.000
Waist Circumference				
Normal ( WC <90 cm M, WC <80 cm F)	1.00		1.00	
Abdominally Obese (WC ≥ 90 cm M, WC ≥ 80 cm F)	6.2 (3.7–10.4)	0.000	2.2 (1.6–3.0)	0.000
Physical Activity				
Low (<150 min/week)	1.00		1.00	
High ≥150 min/week)	0.3 (0.2–0.6)	0.000	0.7 (0.5–0.9)	0.025
Vegetable/ Fruit intake				
<4–5 Servings/Day	1.00		1.00	
≥4–5 Serving/Day	0.2 (0.2–1.9)	0.167	0.1 (0.04–0.2)	0.000

^a^Bangladesh Taka 76.00 = USD 1.00

**Fig. 2:**
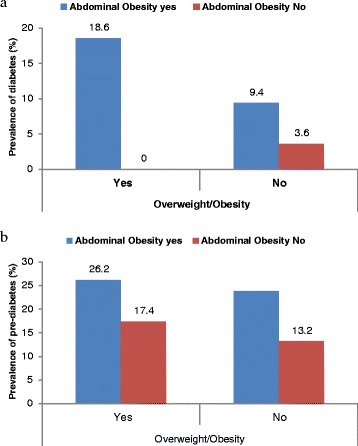
**a** Distribution of diabetes by adiposity indicators. Abdominal obesity was defined as waist circumference ≥0.90 cm for Males, ≥0.85 cm for Females. None of the overweight/obese individual was without abdominal obesity although over 9 % non-overweight/obese individuals had abdominal obesity. **b** Distribution of pre-diabetes by adiposity indicators. Abdominal obesity was defined as waist circumference ≥0.90 cm for Males, ≥0.85 cm for Females

The regression findings showed that, age (>40 y), urban residence, overweight (BMI ≥25 kg/m^2^), abdominal obesity and high WHR were significantly associated with higher probability of having undiagnosed diabetes (Table 3). Compared to the 20–39 year age group, those in the 40–59 years and 60 years or older age groups had nearly three and eight times the risk of un-diagnosed diabetes, respectively. Urban residence was associated with nearly 2.5 fold increased risk of diabetes. Overweight, abdominal obesity and high waist to hip ratio were associated with 2.2, 3.3 and 55.5 fold greater risk of undiagnosed diabetes, respectively. On the otherhand age group 40–59, female gender, secondary education, abdominal obesity and high WHR were significantly associated with increased probability of pre-diabetes (Table 4). Diabetes prevalence was nearly six times higher among overweight participants with abdominal obesity compared to normal weight non-abdominally obese idividuals (Fig. 2a). However, non-overweight individuals with abdominal obesity had three times higher prevalence of diabetes than those without abdominal obesity. Presence of abodominal obesity was also associated with higher prevalence of pre-diabetes in both normal and overweight individuals (Fig. 2b).

**Table 3:** Determinants of diabetes in adults in rural and urban Bangladesh

Value label	Unadjusted		Adjusted (model 1)^a^		Adjusted (Model 2)^a^		Adjusted (Model 3)^a^	
	OR ( 95 % CI)	*p* value	OR ( 95 % CI)	*p* value	OR ( 95 % CI)	*p* value	OR ( 95 % CI)	*p* value
Age								
20–39	1.00		1.00		1.00		1.00	
40–59	2.4 (1.4–4)	0.001	2.9 (1.7–5.2)	0.000	2.7 (1.5–4.8)	.001	2.7 (1.5–4.8)	0.001
60/above	8.4 (3.1–22.2)	0.000	8.1 (2.8–23.8)	0.000	7.6 (2.6–22.5)	.000	5.9 (2.1–17.5)	0.001
Sex								
Male	1.00		1.00		1.00		1.00	
Female	0.8 (0.5–1.3)	0.381	0.9 (0.5–1.6)	0.699	0.8 (0.4–1.4)	.391	1.3 (0.7–2.2)	0.421
Area								
Rural	1.00		1.00		1.00		1.00	
Urban	5.9 (3.5–9.9)	0.000	2.5 (1.02–5.9)	0.045	2.5 (1.1–6.0)	.038	2.8 (1.2–6.6)	0.017
Education								
Illiterate	1.00		1.00		1.00		1.00	
Primary	0.7 (.2–1.8)	0.414	0.6 (0.2–1.6)	0.273	0.5 (0.2–1.5)	.233	0.6 (0.2–1.6)	0.296
Secondary	3.5 (1.8–6.7)	0.000	1.5 (0.7–3.3)	0.303	1.4 (0.6–3.0)	.438	1.6 (0.7–3.5)	0.239
Post-secondary	2.8 (1.4–5.9)	0.003	0.8 (0.3–2.1)	0.681	0.8 (0.3–1.9)	.568	0. 8 (0.3-2.1	0.694
Income (Taka/month)^b^								
Low: ≤6,000	1.00		1.00		1.00		1.00	
Medium: 6,001–12,000	3.2 (1.5–6.7)	0.002	1.5 (0.6–3.4)	0.359	1.4 (0.6–3.4)	.411	1.4 (0.6–3.3)	0.410
High: >12,000	6.3 (3.2–12.3)	0.000	1.6 (0.7–3.9)	0..293	1.6 (0.6–3.9)	.339	1.6 (0.7–3.9)	0.296
Body Mass Index (BMI)								
Normal (18.50–24.99 kg/m^2^)	1.00		1.00					
Underweight (≤18.49 kg/m^2^)	0.3 (0.1–0.9)	0.046	0.5 (0.1–1.5)	0.202				
Overweight (≥25 kg/m^2^)	3.5 (2.2–5.8)	0.000	2.2 (1.3–3.9)	0.006				
Waist Circumference								
Normal (<0.90 M,<0.85 F)	1.00				1.00			
Abdominally Obese (≥0.90 M, ≥0.85 F)	5.6 (3.4–9.2)	0.000			3.3 (1.8–6.0)	.000		
Waist-to-Hip ratio								
Normal ( WC <90 cm M, WC <80 cm F)	1.00						1.00	
High (WC ≥ 90 cm M, WC ≥ 80 cm F)	9.4 (4.6–18.9)	0.000					5.6 (2.7–11.9)	0.000
Physical Activity								
High ≥150 min/week)	1.00		1.00		1.00		1.00	
Low (<150 min/week)	3.4 (1.8–6.5)	0.000	1.1 (0.5–2.6)	0.843	1.1 (0.5–2.6)	.847	0.9 (0.4–2.2)	0.906

^a^Three separate models for three different adiposity indicators, BMI, Waist Circumference and Waist to Hip Ratio as they are highly correlated and essentially indicate body fatness

^b^Bangladesh Taka 76 = USD 1.00

**Table 4:** Determinants of prediabetes in adults in rural and urban Bangladesh

Value label	Unadjusted		Adjusted (model 1)^a^		Adjusted (Model 2)^a^		Adjusted (Model 3)^a^	
	OR ( 95 % CI)	*p* value	OR ( 95 % CI)	*p* value	OR ( 95 % CI)	*p* value	OR ( 95 % CI)	*p* value
Age								
20–39	1.00		1.00		1.00		1.00	
40–59	1.3 (0.9–1.8	0.110	1.6 (1.1–2.2)	0.006	1.6 (1.1–2.2)	0.010	1.5 (1.1–2.1)	0.012
60/above	1.8 (0.7–4.7)	0.225	2.1 (0.8–5.6)	0.161	2.0 (0.7–5.5)	0.169	1.8 (0.7–4.9)	0.249
Sex								
Male	1.00		1.00		1.00		1.00	
Female	1.3 (0.9–1.8)	0.170	1.5 (1.0–2.2)	0.046	1.4 (0.9–2.0)	0.096	1.7 (1.2–2.5)	0.008
Area								
Rural	1.00		1.00		1.00		1.00	
Urban	2.01(1.47–2.72)	0.000 ^a^	1.4 (0.8–2.2)	0.220	1.4 (0.8–2.3)	0.205	1.5 (0.9–2.5)	0.114
Education								
Illiterate	1.00		1.00		1.00		1.00	
Primary	1.3 (0.8–2.2)	0.236	1.3 (0.8–2.1)	0.328	1.3 (0.8–2.0)	0.361	1.3 (0.8–2.1)	0.320
Secondary	2.1 (1.4–3.1)	0.001	1.7 (1.0–2.8)	0.034	1.6 (1.0–2.7)	0.047	1.7 (1.1–2.8)	0.028
Post-secondary	2.2 (1.4–3.5)	0.001	1.8 (0.9–3.3)	0.068	1.7 (0.9–3.2)	0.079	1.8 (0.9–3.3)	0.064
Income (Taka/month)^b^								
Low: ≤6,000	1.00		1.00		1.00		1.00	
Medium: 6,001–12,000	1.8 (1.2–2.6)	0.003	1.3 (0.8–1.9)	0.298	1.3 (0.8–1.9)	0.284	1.3 (0.8–1.9)	0.286
High: >12,000	2.1 (1.5–3.1)	0.000	0.8 (0.5–1.3)	0.738	1.1 (0.7–1.8)	0.761	1.1 (0.7–1.8)	0.711
Body Mass Index (BMI)								
Normal (18.50–24.99 kg/m^2^)	1.00		1.00					
Underweight (≤18.49 kg/m^2^)	0.6 (0.4–1.03)	0.067	.8 (0.5–1.3)	0.298				
Overweight (≥25 kg/m^2^)	1.8 (1.3–2.5)	0.000	1.4 (0.9–2.0)	0.073				
Waist Circumference								
Normal (<0.90 M,<0.85 F)	1.00				1.00			
Abbdominally Obese (≥0.90 M, ≥0.85 F)	2.2 (1.6–3.0)	0.000			1.7 (1.2–2.4)	0.006		
Waist-to-Hip ratio								
Normal ( WC <90 cm M, WC <80 cm F)	1.00						1.00	
High (WC ≥ 90 cm M, WC ≥ 80 cm F)	1.9 (1.4–2.6)	0.000					1.6 (1.2–2.3)	0.005
Physical Activity								
High ≥150 min/week)	1.00		1.00		1.00		1.00	
Low (<150 min/week)	1.5 (1.0–2.0)	0.028	1.1 (0.7–1.7)	0.567	1.1 (0.4–1.2)	0.564	1.2 (0.8–1.8)	0.455

^a^Three separate models for three different adiposity indicators, BMI, Waist Circumference and Waist to Hip Ratio as they are highly correlated and essentially indicate body fatness

^b^Bangladesh Taka 76 = USD 1.00

## Discussion

We measured the determinants and the hidden burden of undiagnosed diabetes and pre-diabetes in urban and rural settings using rigorous diagnostic procedures and identified high risk groups. Our study shows that the age 40 years or older and those with abdominal obesity or overweight has the highest probability of undiagnosed diabetes. In a resource-poor setting like Bangladesh the cost for total population screening would be prohibitively high however, targeting high-risk groups defined by a combination of age (≥40 years abdominal obesity, or overweight can be a first step in diabetes screening and prevention in Bangladeshi population.

Several previous studies in Bangladesh reported prevalence of diabetes between 2.1 %–2.3 % in rural [21, 22], 4.1 % in suburban [23] and around 8.3 % in urban populations [21, 24, 25]. A recent meta-analysis of studies on diabetes in Bangladesh reported an increasing trend of diabetes prevalence since the mid-1990s [4]. The prevalece of diabetes we observed in the rural population is consistent with previous studies, but we found a much higher prevalence of diabetes in urban populations than previously reported with the exception of a recent study in urban Dhaka which reported 35 % study subjects with diabetes [26]. This exceptionally high prevalence might be explained by the older age, high proportion of overweight and obese participants and also accounting for both undiagnosed and already known diabetes cases included in that study. The higher prevalence of diabetes in urban participants in this study is likely attributable to higher prevalence of overweight and abdominal obesity and lower physicaly activity among urban participants.

A number of studies in Bangladesh also reported prevalence of pre-diabetes, either based on fasting blood glucose [16, 27–30] or using OGTT criteria [21, 23, 31, 32] although studies using only fasting blood glucose fail to capture all individuals with prediabetes . We also observed higher prevalence of pre-diabetes, particularly among urban participants, than rural. Altogether one third of urban and 16 % of rural population have dysglycaemia suggesting a disproportionately higher burden in urban populations. The prevalence of prediabetes is generally much higher than diabetes in Bangladesh. Recent report from a national survey based on fasting blood glucose criterion showed that nearly 22 % of adult Bangladeshi have prediabetes [27]. Our data and other evidence suggest that prediabetes is a huge unrecognized problem but offers an urgent opportunity for preventive interventions. Evidence shows that lifestyle modifications can prevent 30 to 67 % of diabetes among individuals with prediabetes through lifestyle modification in different settings [13, 33–35]. Without change there is a projected doubling of diabetes population in Bangladesh up to 16.8 million by 2030 [3]. However, this high burden of disease may be slowed down or prevented if effective interventions for pre-diabetes are undertaken.

Low awareness in terms of diabetes status and control among individuals with diabetes is a global phenomenon and a major barrier to effective glycaemic control. The high prevalence of undiagnosed diabetes observed in this study indicates poor awareness among individuals with diabetes in Bangladesh. The recent Bangladesh National NCD Risk Factor Survey data showed that only 2.2 % of adults reported having diabetics [17], which indicates a very low level of awareness about diabetes in this population. Another more recent publication reported that only 41 % of individuals with diabetes were aware of their condition [16]. A similar high level of unawareness has also been reported from mainland China and Hong Kong where two-thirds and one-half of individuals with diabetes are unaware, respectively [36]. Low awareness about diabetes has also been reported among Black and Hispanic populations in the United Stated [37].

We found nearly a five-fold difference in undiagnosed diabetes between urban and rural settings. The urban participants had higher overweight and obesity scores, higher prevalence of abdominal obesity, lower physical activity, and lower consumption of vegetables, all of which are established risk factors for diabetes. Lower prevalence of these risk factors in the rural cohort may indicate protective lifestyle for diabetes and other chronic non-communicable diseases. The lower BMI, lower abdominal obesity, and higher physical activity of rural population, make them less likely to have insulin resistance [38] and therefore less likely to undergo a rapid transition from pre-diabetes to diabetes. Undiagnosed pre-diabetes is 1.6 times higher in the urban than rural participants. Higher prevalence and an increasing trend in diabetes prevalence in urban population have been reported in India recently [39].

In this study, as can be expected, age is confirmed as a significant predictor of undiagnosed diabetes. However, this association was increasingly stronger for the older age groups. The American Diabetes Association (ADA) suggests screening for diabetes and prediabetes in asymptomatic people in adults of any age who are overweight or obese (BMI ≥25 kg/m2) and who have one or more additional risk factors for diabetes, such as physical inactivity, first degree relatives with diabetes, high-risk race, hypertension, hyperlipidaemia among others [40]. However, most of the suggested risk factors data may not be routinely available in low-income and developing countries like Bangladesh.

Diabetes is one of the most important cardiovascular disease risk factors, and will be increasingly important as global urbanization continues [1]. Diabetes is also a major modifier/predictor of other CVD risk factors. Diabetes and pre-diabetes have implications for the treatment of hypertension when they coexist [41, 42]. Often more that half of diabetes patients have hypertension [43]. As the diabetes population in Bangladesh rapidly increases in the coming decades [3], it will be accompanied by an increased burden of cardiovascular disease unless improved prevention, case detection and treatment are implemented now.

Adiposity indicators such as BMI, WC, and WHR are well known risk factors for diabetes . A meta-analysis of published literature also showed an 88 % increase in the relative risk of diabetes associated with each one standard deviation increase in BMI, WC or WHR [44]. The burden of obesity is still considered to be low in Bangladesh but our data suggest it is a significant problem in urban middle-class people where overweight and abdominal obesity exceed 58 and 62 % respectively [26] A recent report from Bangladesh showed a sharp rise in the proportion of overweight and obesity in adults increased from 3.66 to 16.94 % between 1992 and 2011 [45]. Our findings showed that both overweight and abdominal obesity are associated with higher risk of diabetes and pre-diabetes and those who had both overweight and abdominal obesity had the greatest risks. In general the South-Asian populations are known to have some special characteristics such as higher fat mass for any given BMI as compared to Caucasians and abdominal obesity is prevalent among many people without BMI based obesity [46]. This is the first study to our knowledge which looked at the independent and combined effect of overweight with abdominal obesity as strong recommendation for population based screening in Bangladeshi population.

Unhealthy diet and physical inactivity are major determinants of most chronic diseases including diabetes (Ref.). Among dietary risk factors, drinking sweet sugary beverages increases the risk [47] while dietary fibre, fruits and vegetable consumption are associated with reduced risk [48]. Major dietary guidelines emphasize eating 4–5 or more servings of fruits or vegetables daily as part of a healthy diet [49]. The recent Bangladesh NCD Risk Factor Survey report concluded that over 98 % of the adult population in Bangladesh had inadequate consumption of fruits and vegetables [17]. The current study also found a negative association between fruit and vegetable consumption and diabetes and pre-diabetes. We also observed a negative association bwteen physical activity of moderate or high intensity (for more than 150 min per week) and undiagnosed diabetes and pre-diabetes. We found urban residents less active than their rural counterparts, which may explain the higher prevalence of diabetes and pre-diabetes among urban population. Apart from different lifestyle adaptations, inadequate open space and the increased use of motorized transportation are also among the major barriers to physical activity in the urban Bangladesh.

Although this study has some strengths, it is worthwhile to mention some limitations of the study as well. The major strength of this study is that it used OGTT instead of single measure of fasting glucose concentration which captured diabetes and prediabetes including both IFG and IGT in rural and urban settings. Screening based on single fasting blood glucose criteria suffer lower sensitivity in detecting diabetes as well as prediabetes in all individuals [50]. In this study female participants were over-represented (over 2/3rd) but a lower participation of males was mainly due to their work schedule over the day. The study was conducted in one urban and one rural locations and all the available, eligible consenting participants were included in the study therefore it can not be claimed as a nationally reporesentative study and the results may need careful interpretation However, the characteristics of the study participants are comparable with other similar studies in Bangladesh (ref.). We used finger prick blood and HemoCue ™ method instead of venous blood and laboratory determination of plasma glucose concentration. However, HemoCue provided a validated plasma equivalent reading from finger prick blood.

## Conclusion

The burden of undiagnosed diabetes and pre-diabetes is enormously high in Bangladesh, especially in the urban population, and is related to overweight and abdominal obesity. Aggressive screening would be desirable to identify the hidden levels of diabetes and pre-diabetes, but that might not be feasible in Bangladesh considering the socioeconomic conditions. However, preventive interventions should receive the highest priority to halt the diabetes epidemic and avoid prohibitive treatment costs of diabetes. Our findings suggest population-based screening of people aged 40 years and older with measurement of weight and abdominal obesity has the potential to yield high detection of dysglycaemic conditions and prevent premature onset of diabetes and diabetes-related complications. Further investigation is needed to understand the disproportionately higher burden of diabetes in the urban middle-class population of Bangladesh.
